# Poster Session II A327 INFLUENCE OF *IN UTERO* CANNABIS SMOKE EXPOSURE ON FETAL GUT-BRAIN AXIS DEVELOPMENT

**DOI:** 10.1093/jcag/gwaf042.327

**Published:** 2026-02-13

**Authors:** S F D’Amico, C Monaco, J Kasinska, R Brossaud, C M Fricano, J Feeney, T Podinic, S Raha, E Ratcliffe

**Affiliations:** Pediatrics, McMaster University, Hamilton, ON, Canada; Pediatrics, McMaster University, Hamilton, ON, Canada; Pediatrics, McMaster University, Hamilton, ON, Canada; Pediatrics, McMaster University, Hamilton, ON, Canada; The Department of Psychiatry & Behavioural Neurosciences, McMaster University Faculty of Health Sciences, Hamilton, ON, Canada; Pediatrics, McMaster University, Hamilton, ON, Canada; Pediatrics, McMaster University, Hamilton, ON, Canada; Pediatrics, McMaster University, Hamilton, ON, Canada; Pediatrics, McMaster University, Hamilton, ON, Canada

## Abstract

**Background:**

Cannabis legalization in Canada has highlighted the societal relevance of investigating the potential impacts of *in utero* cannabis exposure on fetal development. Recent Canadian market trends show a consistent rise in the concentration of delta-9-tetrahydrocannabinol (Δ^9^-THC), the primary psychoactive constituent, in recreationally available cannabis. The endocannabinoid system plays a key role in shaping the normal development of the gut-brain axis and could potentially be disrupted by *in utero* cannabis exposure. However, the impact of high-Δ^9^-THC cannabis smoke exposure on the developing gut-brain axis remains poorly understood.

**Aims:**

We tested the hypothesis that *in utero* exposure to high-Δ^9^-THC cannabis smoke would influence fetal gut-brain axis development and initiate early meconium descent.

**Methods:**

We developed a “real-world” cannabis smoke inhalation model where timed-pregnant CD-1 mice (n = 23) were exposed daily from embryonic day (E) 0.5 to E18.5 to the smoke from six high-Δ^9^-THC cannabis cigarettes (29.05% Δ^9^-THC) in a custom-built smoking chamber unit. Control dams (n = 14) were restrained and exposed to room air for an equivalent duration. Maternal weight and food intake were monitored during pregnancy (E0.5–E18.5). At E18.5, dams were sacrificed, and fetal biometrics were recorded. Fetal gastrointestinal tracts (stomach–distal colon) were dissected, and meconium descent was quantified in Fiji. Data were averaged per dam, analyzed using Welch’s t-test, and reported as mean ± SD.

**Results:**

Cannabis-exposed dams gained less weight (p < 0.0001) and consumed less food (p < 0.0001) during pregnancy than control dams. Interestingly, while fetal body weight was reduced (p = 0.0005), brain weight did not differ between groups (p = 0.0724), leading to a higher brain-to-body weight ratio in the cannabis-exposed fetuses (p = 0.0092). Lastly, *in utero* high-Δ^9^-THC cannabis exposure did not affect meconium descent, as measured by the furthest distance of migration [mm] from the cecum (Cannabis: 8.73±1.23; Control: 8.91±1.40; n.s.) and the total length of meconium [mm] in the colon (Cannabis: 3.99±1.23; Control: 4.77±1.58; n.s.).

**Conclusions:**

We found that exposure to high-Δ^9^-THC cannabis smoke *in utero* reduced maternal food intake and weight gain. Fetuses from cannabis-exposed dams had lower body weight compared to controls, but brain weight was preserved. Lastly, high-Δ^9^-THC cannabis smoke exposure *in utero* did not affect the extent of meconium descent in the fetal colon. In the context of Canadian cannabis market trends, these findings underscore the need to further explore the influence of *in utero* exposure to high-Δ^9^-THC cannabis smoke on the developing gut-brain axis and subsequent offspring outcomes.

**Funding Agencies:**

CIHRThe Azrieli Foundation

